# Serum peptidome patterns of hepatocellular carcinoma based on magnetic bead separation and mass spectrometry analysis

**DOI:** 10.1186/1746-1596-8-130

**Published:** 2013-08-05

**Authors:** Xia Ying, Su-xia Han, Jun-lan Wang, Xia Zhou, Gui-hua Jin, Long Jin, Hao Wang, Lei Wu, Jianying Zhang, Qing Zhu

**Affiliations:** 1 Department of Medical Oncology, the First Affiliated Hospital of Xi’an Jiaotong University, Xi’an, Shannxi, PR China

**Keywords:** Hepatocellular carcinoma, Chronic hepatitis, Liver cirrhosis, Magnetic beads, Matri-assisted laser desorption/ionization time-of-flight mass spectrometry, Serum biomarkers

## Abstract

**Background:**

Hepatocellular carcinoma (HCC) is one of the most common cancers in the world,and the identification of biomarkers for the early detection is a relevant target. The purpose of the study is to discover specific low molecular weight (LMW) serum peptidome biomarkers and establish a diagnostic pattern for HCC.

**Methods:**

We undertook this pilot study using a combined application of magnetic beads with Matrix-assisted laser desorption/ionization time-of-flight mass spectrometry (MALDI-TOF MS) technique and ClinPro Tools v2.2 to detect 32 patients with HCC, 16 patients with chronic hepatitis (CH), 16 patients with liver cirrhosis (LC) and 16 healthy volunteers.

**Results:**

The results showed 49, 33 and 37 differential peptide peaks respectively appeared in HCC, LC and CH groups. A Supervised Neural Network (SNN) algorithm was used to set up the classification model. Eleven of the identified peaks at m/z 5247.62, 7637.05, 1450.87, 4054.21, 1073.37, 3883.64, 5064.37, 4644.96, 5805.51, 1866.47 and 6579.6 were used to construct the peptides patterns. According to the model, we could clearly distinguish between HCC patients and healthy controls as well as between LC or CH patients and healthy controls.

**Conclusions:**

The study demonstrated that a combined application of magnetic beads with MALDI-TOF MB technique was suitable for identification of potential serum biomarkers for HCC and it is a promising way to establish a diagnostic pattern.

**Virtual slides:**

The virtual slide(s) for this article can be found here: http://www.diagnosticpathology.diagnomx.eu/vs/1503629821958720.

## Background

Primary liver cancer (PLC) is one of the most common digestive cancers. HCC accounts for 90% of PLC [1]. HCC is the fifth most common human cancer, with approximately 750 000 new cases occurring worldwide every year [2]. HCC ranks the third in the annual global cancer mortality rates [3] and the average time from the discovery of symptoms to death is 6–20 months [1,4-6]. In China HCC is one of the leading causes of mortality and morbidity [7]. Surgery, chemotherapy and radiation treatments can be effective therapy, which depending on the stages of cancer and other factors. The high case-fatality rate can be partly attributed to lack of methods to early diagnosis. Early diagnosis and treatment is an effective way to improve patient survival. Serum markers are thought to be simple and accurate tools for HCC diagnosis; However, no ideal biomarker has been found so far. Although alpha fetoprotein (AFP) is the most widely used serum biomarker for HCC diagnosis, its sensitivity (39-64%) and specificity (76-91%) are not optimal [8-10]. In addition, patients with CH or LC may also show elevated AFP levels [11,12]. Furthermore, liver biopsy is an invasive procedure and not suitable for screening of HCC. Therefore, there is a need for the development of more sensitive and specific methods for the early diagnosis of HCC.

Proteomics is currently considered the most powerful tool for the global evaluation of protein expression [13] since protein deficiency is considered common in cancer patients. Peptides in human serum may have a correlation with the physiologic and pathologic processes [14]. Low molecular weight (LMW) proteins, particularly on peptides smaller than 20 KDa, are expected to yield useful biomarkers for early diagnosis of cancers [15].

Matrix-assisted laser desorption/ionization time-of-flight mass spectrometry (MALDI-TOF MS) can detect peptides with low molecular weights [16,17] and is considered to be a powerful proteomic technology for serum peptide profiling. Magnetic beads (MB) based purification approaches has been developed to capture large amounts of LMW peptides and proteins in biological samples, which are suitable for the next MS analyses [18]. Combined with MALDI-TOF MS technology, MB is more precise, rapid and robust than the traditional two-dimensional (2-D) gel electrophoresis. MB-based platform for proteomic profiling highlights the high sensitivity and reproducibility. This method has been applied to identify biomarkers for diseases such as esophageal carcinoma [19], multiple myeloma [20], lung cancer [21] and breast cancer [22].

In the study, MALDI-TOF MS analysis coupled with MB-WCX were used to detect 32 patients with HCC, 16 patients with CH, 16 patients with LC and 16 healthy volunteers. We aimed to investigate LMW serum protein/peptide biomarkers for HCC and ultimately construct a diagnostic model for improving diagnosis efficiency of HCC.

## Methods

### Reagents and instruments

The Autoflex III MALDI-TOF mass spectrometer, α-cyano-hydroxy cinnamic acid (HCCA), MB-WCX kit and peptide calibration standard were purchased from Bruker Daltonics (Germany).

### Patients and sample collection

The study was approved by the Ethics Committee and the Human Research Review Committee of Xi’an Jiaotong University. A total of 80 serum samples were collected, of which 32 were collected from HCC patients, 16 from patients with CH, 16 from patients with LC and 16 from healthy volunteers as healthy control. The dataset of serum samples and their donors are listed in Table 1, while the patients’ information about diagnostics, age, sex and potential cause are listed in Additional file 1. These blood samples were collected in the first Affiliated Hospital of Xi’an Jiaotong University (China), from October 2011 to September 2012. All the HCC patients had been recently diagnosed and pathologically confirmed before they were treated with chemotherapy or radio therapy or surgery. The healthy subjects were those who were hepatitis B surface antigen (HBsAg) negative, and had no evidence for malignant tumor, no chronic diseases, and no dysfunction of vital organs. All blood samples were drawn while the patients and healthy controls were seated and non-fasting. The samples were collected in vacuum tubes, allowed to clot at room temperature for 30 min, and then centrifuged at 3,000 rpm for 5 min. The serum samples were distributed into 300 μL aliquots each and stored at −80°C until analysis.

**Table 1:** Dataset of serum samples and their donors

**Group**	**Sample size**	**Gender**	**Mean age**	**Age range**
HCC	32	27 M/5 F	52.72	26-70
LC	16	11 M/5 F	54.31	40-69
CH	16	11 M/5 F	54.06	42-73
Healthy control	16	11 M/5 F	54.69	43-66

### Sample purification and MALDI-TOF MS analysis

Magnetic beads-based weak cation exchange chromatography (ClinProt^TM^ purification reagent sets of Bruker Daltonics) was used to extract peptides/proteins from the serum samples following the manufacturer’s standard protocol. 10 μl of MB-WCX binding solution and 10 μl of WCX-beads were mixed in a 0.5 ml centrifuge tube. After the mix was thoroughly combined, 5 μl serum sample was added and mixed up and down. Then centrifuge tubes were placed in a magnetic bead separator (MBS) and agitated three times. The beads were collected from the wall of the tubes in the MBS 1 min later. Remove the supernatant carefully by using a pipette and add 100 μl MB-WCX wash buffer to the tubes, which were agitated back and forth in the MBS ten times. The beads were collected from the wall of the tubes, and supernatant was removed carefully. After two washes, 5 μl of MB-WCX elution buffer was added to disperse beads in tubes by pipetting up and down ten times. The beads were collected on wall of tubes for 2 min and the clear supernate were transferred into fresh tubes. 5 ul MB-WCX stabilization solution was added to the fresh tubes and mixed intensively by pipetting up and down. The eluate was then ready for spotting onto MALDI-TOF MS targets and measurement.

To prepare the MALDI target, 1 μL of a mixture containing 10 μL 0.3 g/L α-cyano-4-hydroxy cinnamic acid (HCCA) in 2:1 ethanol/acetone (volume/volume) and 1 μL of the eluate was spotted onto the MALDI AnchorChip TM (Bruker Daltonics, Germany) sample target platform (384 spots) and then the target was air-dried (cocrystallization).

### MS analysis

A linear Autoflex III MALDI-TOF mass spectrometer (Bruker Daltonics, Germany) was used with the following settings: ion source 1, 20.00 kV; ion source 2, 18.40 kV; lens, 7.50 kV; and pulsed ion extraction, 120 ns. Ionization was achieved via irradiation at the laser frequency of 25 Hz. A high gating factor with signal suppression up to 800 Dalton (Da) was used. The mass spectra were recorded in a linear positive mode. Mass calibration was performed using the calibration mixture of the peptides and proteins in the mass range of 1–10 kDa. Three MALDI preparations (MALDI spots) were measured for every MB fraction. For every MALDI spot, 450–550 spectra were quantified (30 laser shots at 15 to 18 different spot positions). The spectra were recorded automatically using the Autoflex Analysis software for the fuzzy-controlled adjustment of the critical instrument settings to generate raw data with optimized quality.

### Data processing with ClinPro tools software

Data analyses were performed using Flex analysis version 3.0 and ClinPro Tools 2.2 (Bruker Daltonics, Germany). ClinPro Tools version 2.2 that uses a standard data preparation workflow including spectra pretreatment, peak picking and peak calculation operation, was employed to recognize peptide patterns. For statistical analysis, a SNN algorithm as implemented in this software suite was used to identify statistically significant differences in protein peaks among the groups analyzed. The protein fingerprint data were analyzed by ClinPro Tools 2.2. Comparisons between HCC, chronic hepatitis, liver cirrhosis and healthy controls were performed with the Wilcoxon test. Statistical significance was assumed when P value was < 0.001.

## Results

### Serum LMW protein profile identification

**Figure 1:**
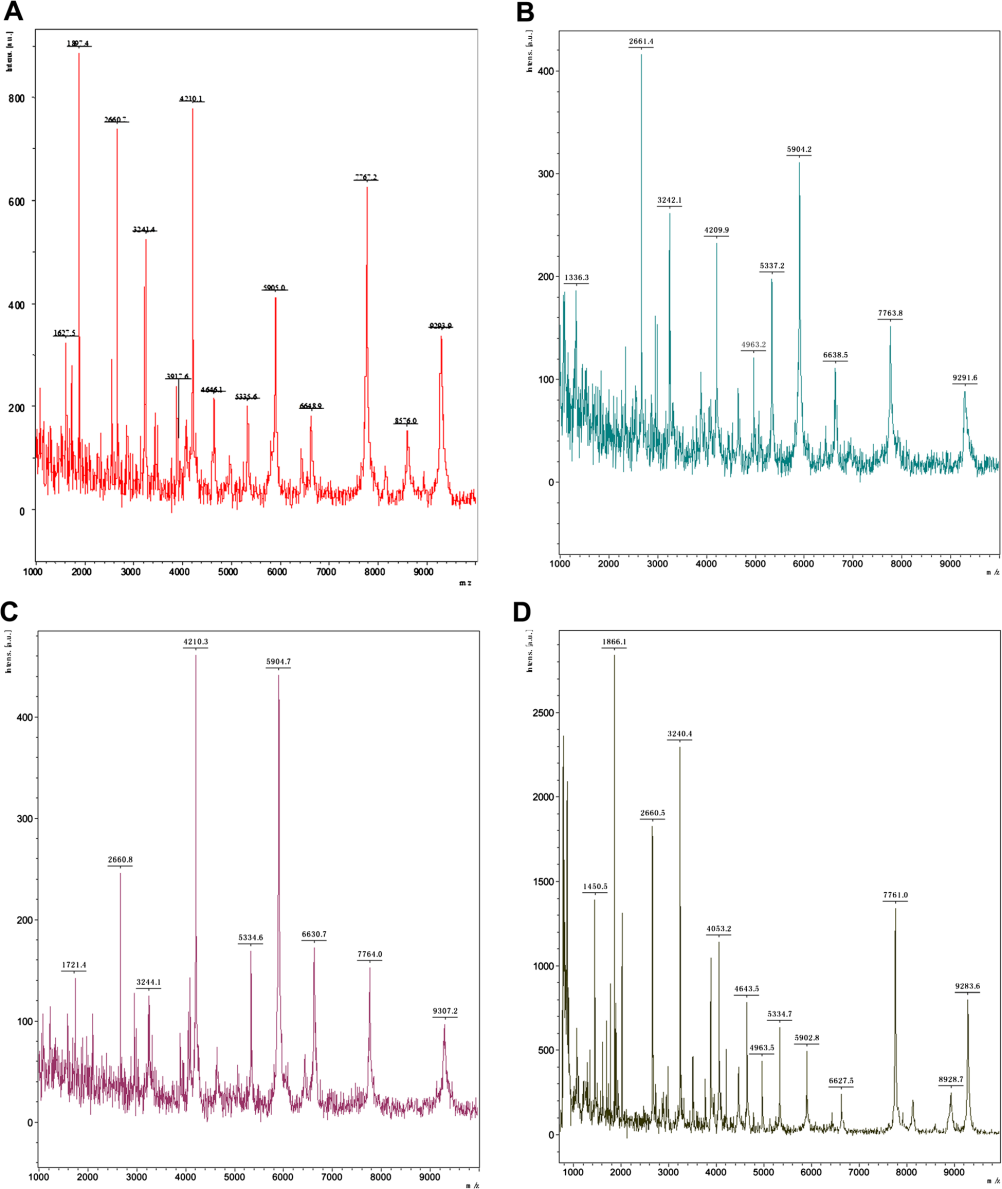
**Representative protein spectrum (1,000-10,000 Da) obtained from 4 group patients. (A)** One sample of hepatocellular carcinoma. **(B)** One sample of liver cirrhosis. **(C)** One sample of chronic hepatitis. **(D)** One sample of healthy control.

MALDI-TOF MS combined with MB-WCX were used in the study to detect LMW protein profile spectra. A total of 80 serum samples from 32 HCC patients, 16 LC patients, 16 CH patients and 16 healthy controls were analyzed (Table 1) and the representative protein profile spectrum of each group was reported as in Figure 1.

### Comparison of mass spectra between HCC, CH, LC and controls

**Figure 2:**
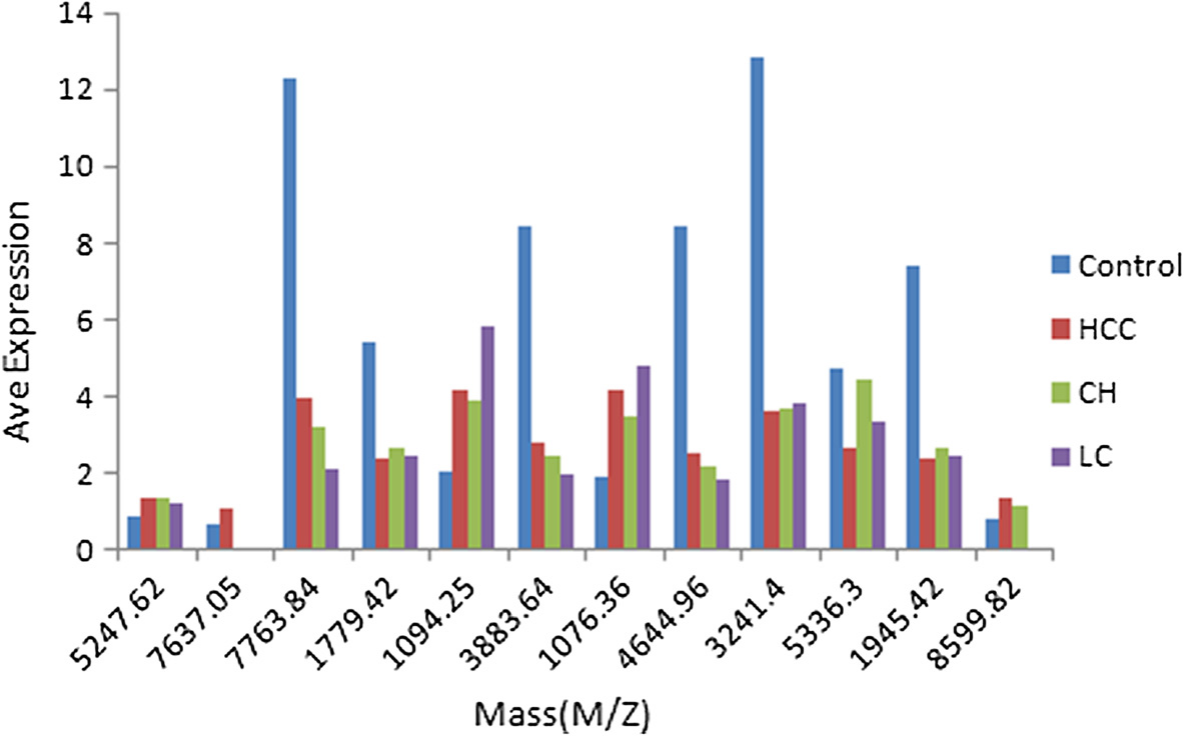
Comparison of the average expression levels at 12 m/z peaks (p < 0.001) between 4 groups.

We analyzed the LMW protein spectra of the three patient groups and healthy controls using a combined application of MB-WCX and MALDI-TOF MS within the mass range from 1 kDa to 10 kDa. Comparing three patient groups with the healthy controls, the study respectively detected 72, 62 and 58 m/z peaks in HCC, LC and CH groups; 49, 33 and 37 of them were significant (p < 0.001) when HCC, LC and CH groups compared with the healthy controls. When detection across all these groups was performed, there were 64 significant peaks. The average expressions of 12 selected peaks were exhibited as shown in Figure 2.

### Analysis of the LMW protein biomarkers and establishment of a diagnostic model for HCC

The protein samples were classified and a diagnostic model was established by using the SNN algorithm in ClinPro Tools software 2.2 to analyze all the detected peaks. The model comprised 11 potential biomarkers (m/z: 5247.62, 7637.05, 1450.87, 4054.21, 1073.37, 3883.64, 5064.37, 4644.96, 5805.51, 1866.47, 6579.6). The peaks at m/z 5247.62, 7637.05, 1073.37, 5064.37, 5805.51 and 6579.6 were up-regulated in the HCC group, while the peaks at m/z 1450.87, 4054.21, 3883.64, 4644.96 and 1866.47 were down-regulated in the HCC group. A combination of these 11 peaks could clearly distinguish between HCC patients and healthy controls as well as between LC or CH patients and healthy controls.

## Discussion

Hepatic tumor has different cell types, such as hepatocellular carcinoma, cholangiocarcinoma, bile duct cystadenocarcinoma, combined hepatocellular and cholangiocarcinoma, hepatoblastoma, undifferentiated carcinoma, hepatic angiomyolipoma, while different biomarkers have been developed and investigated for diagnosis, tumor progression, and prognosis of them [23-26]. It is also well recognized that biomarkers which may play important roles in physiologic and pathologic processes are important for diagnosis, prognosis, and prediction of HCC. Human serum contains LMW protein/peptides, which could be used as biomarker candidates,such as fibrinogen α-chain fibrinogen alpha, albumin and apolipoprotein A1 [27,28].

Compared to genomic approaches, proteomic analysis has the advantage of detecting co-translational and post-translational modifications of proteins which may have important biological functions [29]. MS is one of the most important techniques in proteomic analysis. MALDI-TOF MS is widely used in proteomics biomarker research due to its high sensitivity and high quality in analysis of peptides, proteins and large organic molecules [27-29].

HCC is one of the most common cancers in the world. Although AFP is a widely used serological marker for detection of HCC, its sensitivity and specificity are not optimal and it may also increase in patients with acute and chronic viral hepatitis, liver cirrhosis, and toxic injury [8,9]. Therefore, use of AFP in the screening of early HCC is challenged and a new method for HCC early diagnosis is badly needed.

At present, serum or plasma proteomic analysis has been widely used to compare tumor patients with healthy controls. This technique can also be applied to HCC serum or plasma markers research. Looi KS et al. (2008) applied a proteomic approach (two-dimension gel electrophoresis and liquid chromatography-tandem mass spectrometry) to immune-screen sera from patients with HCC and pre-HCC conditions such as liver cirrhosis and chronic hepatitis as well as sera from normal individuals, and identified 28 HCC-associated tumor antigens, such as heat shock protein 60 (HSP60) and heat shock protein 70 (HSP70) [30]. Mas VR et al. (2009) used Thermo linear ion-trap mass spectrometer (LTQ) coupled with a high performance liquid chromatography electrospray ionization tandem mass spectrometry (HPLC-ESI-MS/MS) and SEQUEST database search algorithms for peptide sequence identification. They found that 18 proteins from HCC patients showed significant changes compared with proteins from patients with HCV-cirrhosis and early HCV-HCC [31].

In the study, serum samples were divided into four groups (HCC, LC, CH and normal controls). MB-WCX was used for purification of LMW proteins/peptides in these serum samples. Peptides profile spectra were detected by MALDI-TOF MS and analyzed by ClinProt Tools software 2.2. We found 49 (HCC group), 33 (LC group) and 37 (CH group) peaks were significantly different from those in healthy controls (p < 0.001). All these peak differences may be associated with pathologic processes, specific immunity response or some risk factors, and may become potential biomarkers in early diagnosis. In addition, when comparing HCC patients with healthy controls, Liu T et al. (2011) reported 9 significant discrimination peaks at m/z 2862.79, 8862.77, 8931.95, 3935.62, 8141.78, 5248.47, 3955.45, 7765.78 and 1944.91 [32], which are close to 2863.11, 8867.91, 8931.25, 3935.27, 8138.19, 5247.62, 3955.8, 7763.84 and 1945.42 detected in the present study. In fact, a single biomarker has an inherent specificity and sensitivity that can not be improved, but multiple biomarkers can be combined to achieve improved clinical performance. In the study a diagnostic model generated by SNN algorithm analysis comprised 11 potential biomarkers (m/z: 5247.62, 7637.05, 1450.87, 4054.21, 1073.37, 3883.64, 5064.37, 4644.96, 5805.51, 1866.47 and 6579.6). Using this established diagnostic model, HCC, LC and CH patients could be distinguished from healthy controls; However, the HCC, LC or CH group could not be accurately identified using the model probably due to the small number of the patients enrolled in the study. Therefore, a large number of patients should be enrolled in the further study to establish a diagnostic model that is effective enough to distinguish among the three diseases.

Now, we are making investigation on these 11 m/z peaks that are co-expressed in hepatocellular carcinoma and other liver-related diseases in order to identity and characterize these biomarkers. In future research, we will validate them by western blot or ELISA technology and try to find a correlation with histopathology findings and cancer staging. Furthermore, we will reveal the biological roles of these proteins/peptides in the pathogenesis and processes of HCC.

## Conclusions

The study confirmed that the combined application of magnetic beads, MALDI-TOF MS technique and ClinPro Tools 2.2 are suitable for LMW serum proteomic analysis and it is a promising way to establish a diagnostic pattern. A limitation of the study is the small number of patients enrolled in the study. A study with a large patient cohort is needed to generate more objective and conclusive results. With a large number of patients, different HCC stages and impact factors could be analyzed and more objective evaluation could be made on ClinProt technology applied in HCC.

## Consent

Written informed consent was obtained from the patient’s guardian/parent/next of kin for the publication of this report and any accompanying images.

## Abbreviations

PLC: Primary liver cancer; AFP: Alpha-fetoprotein protein; HCC: Hepatocellular carcinoma; LC: Liver cirrhosis; CH: Chronic hepatitis; MB-WCX: Magnetic beads-based weak cation exchange chromatography; MB: Magnetic beads; MBS: Magnetic bead separator; MALDI-TOF MS: Matrix-associated laser desorption and ionization time-of-flight mass spectrometry; m/z: Mass-to-charge ratio; Da: Dalton; LMW: Low molecular weight; HCCA: α-cyano-hydroxy cinnamic acid; LTQ: Linear ion-trap mass spectrometer; HPLC-ESI-MS/MS: High performance liquid chromatography electrospray ionization tandem mass spectrometry; HSP: Heat shock protein; SNN: Supervised neural network.

## Competing interests

All authors declare that they have no competing interests.

## Authors’ contributions

QZ, SH and XY conceived this project. XY, JW, XZ, LJ, GJ, HW and LW collected all the serum samples. XY carried out all the experiments and analyses as principal investigator. The paper was wrote by XY also. QZ, JZ and SH supervised this project. All authors read and approved of the final manuscript.

## Supplementary Material

Additional file 1 **Patients’ information of each group.** Shows the diagnostics of the included patients together with the patients' age, sex and potential cause. Click here for file
