# Supplementary material for: Serum peptidome patterns of hepatocellular carcinoma based on magnetic bead separation and mass spectrometry analysis
Source: Diagn Pathol. 2013 Aug 5;8:130. doi: 10.1186/1746-1596-8-130 (PMC3751178; doi:10.1186/1746-1596-8-130)
Supplement: Additional file 1 — Patients’ information of each group. Shows the diagnostics of the included patients together with the patients' age, sex and potential cause. [file 1746-1596-8-130-S1.doc]

| Hepatocellular carcinoma (HCC group) | | | | | |
| --- | --- | --- | --- | --- | --- |
| NO. | Number of patients | Diagnostic methods | age | sex | Potential cause |
| 1 | 392 | surgery | 44 | female | Hepatitis B |
| 2 | 383 | Surgery | 61 | female | Hepatitis B |
| 3 | 363 | surgery | 50 | male | Hepatitis B |
| 4 | 348 | surgery | 48 | male | Hepatitis B |
| 5 | 346 | surgery | 54 | male | Hepatitis B |
| 6 | 341 | surgery | 42 | male | Hepatitis B |
| 7 | 337 | surgery | 48 | male | Hepatitis B |
| 8 | 334 | surgery | 56 | male | Hepatitis B |
| 9 | 332 | biopsy | 66 | female | Hepatitis B |
| 10 | 330 | surgery | 50 | male | Hepatitis B |
| 11 | 325 | surgery | 58 | female | Hepatitis C |
| 12 | 328 | biopsy | 55 | female | Hepatitis B |
| 13 | 329 | surgery | 70 | male | Hepatitis B |
| 14 | 310 | surgery | 46 | male | Hepatitis B |
| 15 | 304 | biopsy | 53 | male | Hepatitis B |
| 16 | 301 | surgery | 68 | male | Hepatitis B |
| 17 | 289 | surgery | 26 | male | Hepatitis B |
| 18 | 277 | surgery | 57 | male | Hepatitis B |
| 19 | 276 | surgery | 47 | male | alcohol |
| 20 | 268 | biopsy | 49 | male | Hepatitis B |
| 21 | 257 | surgery | 69 | male | Hepatitis B |
| 22 | 254 | surgery | 57 | male | Hepatitis C |
| 23 | 243 | surgery | 42 | male | Hepatitis B |
| 24 | 244 | surgery | 58 | male | Hepatitis B |
| 25 | 220 | surgery | 47 | male | Hepatitis B |
| 26 | 210 | surgery | 70 | male | Hepatitis B |
| 27 | 203 | surgery | 46 | male | Hepatitis B |
| 28 | 204 | Biopsy+surgery | 52 | male | Hepatitis B |
| 29 | 205 | surgery | 50 | male | Hepatitis B |
| 30 | 193 | surgery | 43 | male | Hepatitis B |
| 31 | 197 | surgery | 48 | male | Hepatitis B |
| 32 | 184 | surgery | 57 | male | Hepatitis B |

| Liver cirrhosis (LC group) | | | | | |
| --- | --- | --- | --- | --- | --- |
| NO. | Number of patients | Diagnostic methods | age | sex | Potential cause |
| 1 | 14 | Clinical diagnosis | 56 | female | Hepatitis C |
| 2 | 60 | Clinical diagnosis | 55 | male | Hepatitis B |
| 3 | 68 | Clinical diagnosis | 54 | female | Hepatitis B |
| 4 | 72 | Clinical diagnosis | 46 | male | Hepatitis B |
| 5 | 115 | Clinical diagnosis | 50 | male | Hepatitis B |
| 6 | 119 | Clinical diagnosis | 50 | male | Hepatitis B |
| 7 | 124 | Clinical diagnosis | 66 | female | Hepatitis B |
| 8 | 126 | Clinical diagnosis | 55 | male | Hepatitis B |
| 9 | 132 | Clinical diagnosis | 57 | male | Hepatitis B |
| 10 | 160 | Clinical diagnosis | 40 | male | Hepatitis B |
| 11 | 183 | Clinical diagnosis | 49 | male | Hepatitis B |
| 12 | 188 | Clinical diagnosis | 59 | female | Hepatitis B |
| 13 | 189 | Clinical diagnosis | 49 | male | Hepatitis B |
| 14 | 190 | Clinical diagnosis | 69 | male | Hepatitis B |
| 15 | 219 | Clinical diagnosis | 69 | male | Hepatitis B |
| 16 | 232 | Clinical diagnosis | 45 | female | Hepatitis B |

| Chronic hepatitis (CH group) | | | | | |
| --- | --- | --- | --- | --- | --- |
| NO. | Number of patients | Diagnostic methods | age | sex | Hepatitis type |
| 1 | 2 | Clinical diagnosis | 65 | female | Hepatitis C |
| 2 | 32 | Clinical diagnosis | 46 | male | Hepatitis B |
| 3 | 36 | Clinical diagnosis | 47 | male | Hepatitis B |
| 4 | 45 | Clinical diagnosis | 56 | female | Hepatitis B |
| 5 | 47 | Clinical diagnosis | 65 | male | Hepatitis B |
| 6 | 51 | Clinical diagnosis | 58 | male | Hepatitis B |
| 7 | 57 | Clinical diagnosis | 46 | female | Hepatitis B |
| 8 | 99 | Clinical diagnosis | 42 | male | Hepatitis B |
| 9 | 107 | Clinical diagnosis | 44 | male | Hepatitis B |
| 10 | 115 | Clinical diagnosis | 54 | male | Hepatitis B |
| 11 | 117 | Clinical diagnosis | 57 | female | Hepatitis C |
| 12 | 118 | Clinical diagnosis | 47 | male | Hepatitis B |
| 13 | 165 | Clinical diagnosis | 73 | male | Hepatitis B |
| 14 | 172 | Clinical diagnosis | 51 | male | Hepatitis B |
| 15 | 194 | Clinical diagnosis | 49 | male | Hepatitis B |
| 16 | 199 | Clinical diagnosis | 65 | female | Hepatitis C |

| NO. | Number of health controls | age | sex |
| --- | --- | --- | --- |
| 1 | 27 | 53 | male |
| 2 | 72 | 47 | male |
| 3 | 84 | 45 | female |
| 4 | 86 | 64 | male |
| 5 | 96 | 59 | female |
| 6 | 100 | 52 | male |
| 7 | 115 | 66 | female |
| 8 | 119 | 72 | male |
| 9 | 120 | 59 | female |
| 10 | 125 | 54 | female |
| 11 | 135 | 57 | male |
| 12 | 136 | 43 | male |
| 13 | 147 | 47 | male |
| 14 | 148 | 48 | male |
| 15 | 152 | 52 | male |
| 16 | 181 | 57 | male |
